# Morphological Plasticity of LiCl Clusters Interacting with Grignard Reagent in Tetrahydrofuran

**DOI:** 10.1021/jacs.3c04238

**Published:** 2023-07-20

**Authors:** Marinella de Giovanetti, Sondre H. Hopen Eliasson, Abril C. Castro, Odile Eisenstein, Michele Cascella

**Affiliations:** † Department of Chemistry and Hylleraas Centre for Quantum Molecular Sciences, University of Oslo, P.O. Box 1033 Blindern, 0315 Oslo, Norway; ‡ ICGM, University Montpellier, CNRS, ENSCM, 34090 Montpellier, France

## Abstract

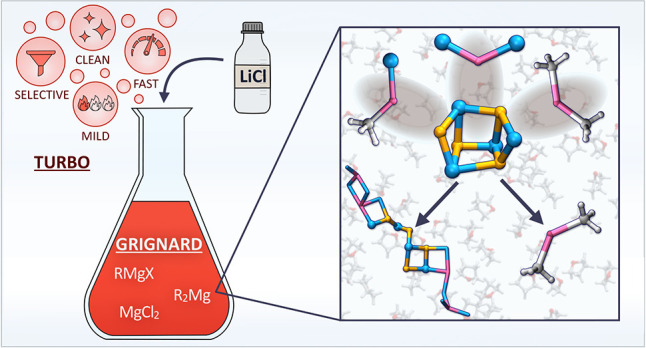

Ab initio molecular dynamics simulations are used to explore tetrahydrofuran (THF) solutions containing pure LiCl and LiCl with CH_3_MgCl, as model constituents of the turbo Grignard reagent. LiCl aggregates as Li_4_Cl_4_, which preferentially assumes compact cubane-like conformations. In particular, an open-edge pseudotetrahedral frame is promoted by solvent-assisted Li–Cl bond cleavage. Among the Grignard species involved in the Schlenk equilibrium, LiCl prefers to coordinate MgCl_2_ through μ_2_-Cl bridges. Using a 1:1 Li:Mg ratio, the plastic tetranuclear LiCl cluster decomposes to a highly solvated mixed LiCl·MgCl_2_ aggregate with prevalent Li–(μ_2_-Cl)_2_–Mg rings and linear LiCl entities. The MgCl_2_-assisted disaggregation of Li_4_Cl_4_ occurs through transient structures analogous to those detected for pure LiCl in THF, also corresponding to moieties observed in the solid state. This study identifies a synergistic role of LiCl for the determination of the compounds present in turbo Grignard solutions. LiCl shifts the Schlenk equilibrium promoting a higher concentration of dialkylmagnesium, while decomposing into smaller, more soluble, mixed Li:Mg:Cl clusters.

The association of Grignard reagents to LiCl (turbo Grignard reagents) constitutes a pivotal step forward in main-group organometallic chemistry. This combination yields more accessible Grignard-like compounds that produce more powerful and controllable reactions.^1−4^ Despite their undisputed success, the enhancing role of lithium salts in the Grignard reaction, one of many examples of the synergistic effect of lithium in heterometallic systems,^5−13^ has remained ununderstood. Better insight is hampered by the difficulty in determining the structures of these compounds in solution. The Grignard reagent forms a diversity of species bearing various ligands coexisting in fast equilibria, as exemplified by the Schlenk equilibrium,^14^ and whose exact composition depends on the experimental conditions.^15−17^ Likewise, multiple forms of LiCl have been observed in the solid state. The detected species, putatively existing also in solution, comprise molecular monomeric,^18^ dimeric,^19−21^ fused ring,^22^ and cubane moieties^23,24^ or polymeric and periodic aggregates,^25,26^ including both neutral and charged states.^27,28^ Combining LiCl with Grignard reagent only obscures the situation further. Attempts to determine the nature of turbo Grignard reagents suffer from the fact that experimental characterization, by crystallization or spectroscopic methods, requires experimental conditions different from those at which reactions occur. Thus, for decades, no neutral Li:Mg moiety was reported in either the gas phase or solid state, but this was not conclusive on their existence in solution.^29,30^ Indeed, very recently tBu_2_MgClLi(THF)_4_ was isolated and characterized by X-ray crystallography, and its presence in solution was assessed by NMR spectroscopy, its monomeric form possibly being promoted by the bulky tBu groups.^31^

In view of the experimental challenges, computational modeling offers a valuable complementary tool. Specifically, ab initio molecular dynamics (AIMD) simulations^32^ are well-suited for studying highly dynamic systems in solution, as shown by their successful use for the characterization of the Schlenk equilibrium involving CH_3_MgCl in tetrahydrofuran (THF) and the related Grignard reaction with acetaldehyde and fluorenone.^33,34^ Here we apply this methodology to shed light on the structural preferences of LiCl in THF and its interaction with a Grignard reagent, represented by CH_3_MgCl and its Schlenk-associated compounds MgCl_2_ and Mg(CH_3_)_2_ (computational details in the Supporting Information).

While the structure of lithium–halide aggregates is debated, there is ample evidence that the four-membered Li_2_X_2_ ring represents an underlying structural motif.^21,23,24,26,35,36^ For this reason, we started from Li_2_Cl_2_ for determining the structural forms of LiCl in THF (Figure 1A). The diamond-shaped ring Li_2_Cl_2_ has two bridging chlorides and two THFs at each lithium, yielding the expected Li_2_Cl_2_(THF)_4_. AIMD provides a solvated structure in satisfactory agreement with that from X-ray diffraction (Table 1), with differences of 1.5–3% for bond distances.

**Figure 1:**
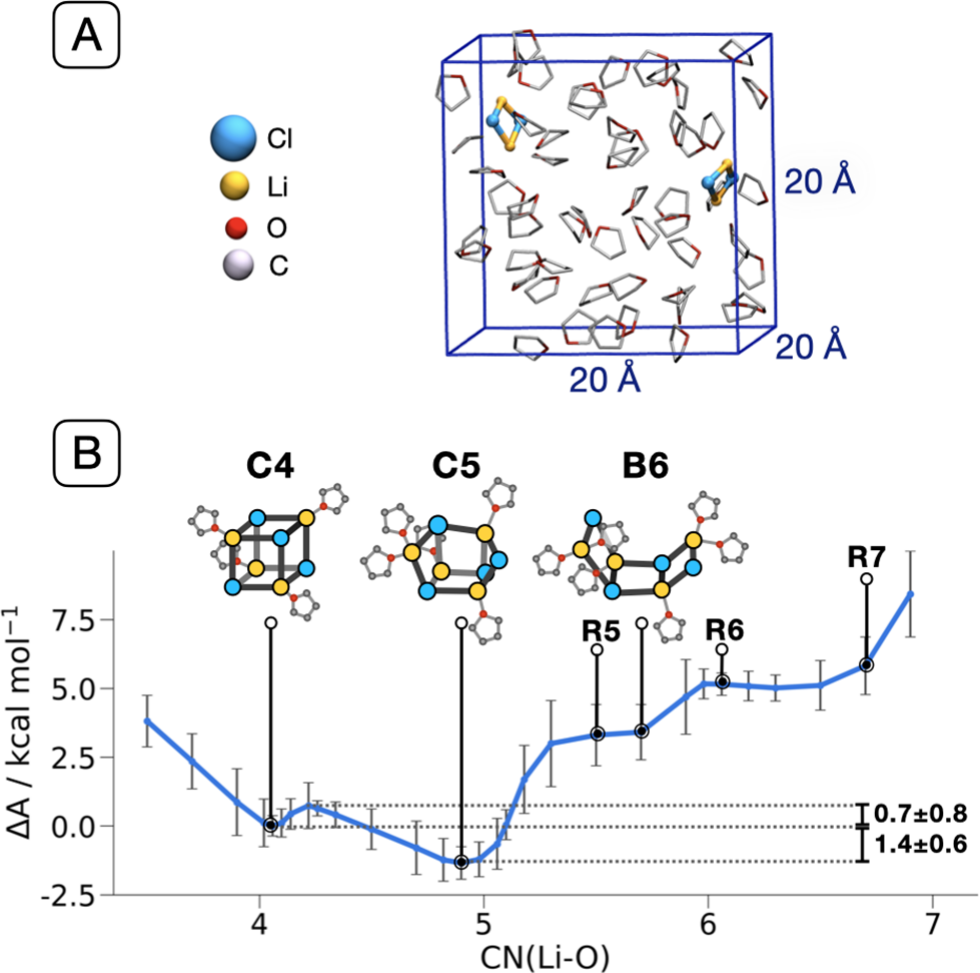
(A) Initial simulation box comprising two Li_2_Cl_2_ entities in THF. Hydrogens are not shown for clarity. (B) Free energy profile (Δ*A*) as a function of the average coordination number between THF and Li (eq S2). **B**, **C**, and **R** stand for boat-, cubane-, and ring-type structures, respectively, followed by the total number of coordinated THFs. See Figure S3 for an extended version showing all structures.

**Table 1:**
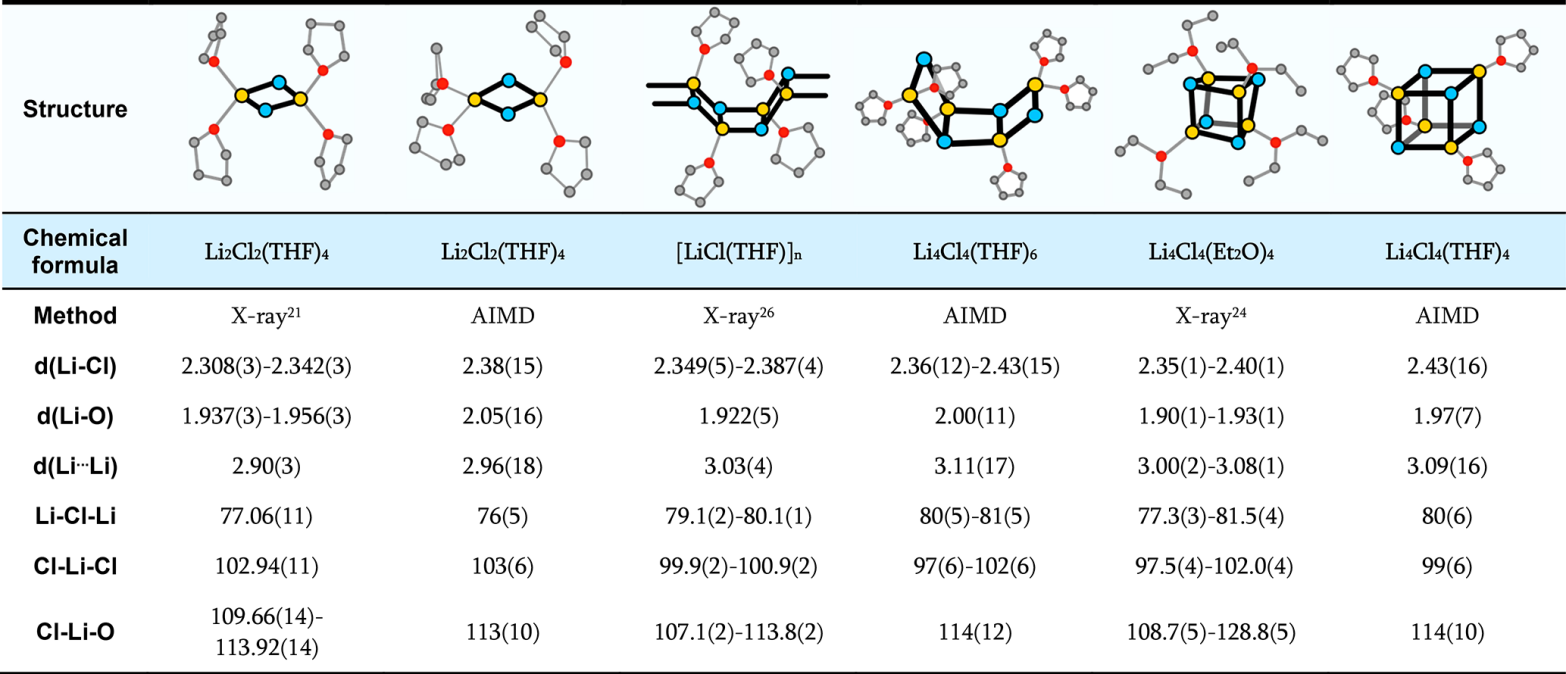
Selected Crystallographic Structures of Li_*n*_Cl_*n*_ with the Corresponding Compounds Found by AIMD in THF^a^

a Distances in Å and angles in deg. Blue = Cl; yellow = Li; red = O; gray = C. Hydrogens are not shown for clarity.

AIMD shows that attraction between two Li_2_Cl_2_(THF)_4_ units starts at a relatively long distance of 8 Å between their respective centers of mass. We observed spontaneous partial desolvation and the formation of two new Li–Cl bonds, yielding Li_4_Cl_4_(THF)_6_ (**B6**, Figure 1B), indicating direct solvent control onto the multimerization of LiCl. **B6** has three condensed four-membered rings in a boat-type structure, with the two external rings in a *cis* arrangement. The lithium atoms of the peripheral rings are solvated by two THFs, while those of the central ring accommodate each a single THF. While a *trans* arrangement cannot be excluded, we note that the structure of **B6** resembles the motif of the THF-solvated LiCl^26^ and LiBr^35^ polymers characterized by X-ray diffraction (Table 1 and Figure S1A).

The Helmholtz free energy (Δ*A*) against THF solvation evidences how **B6** is a metastable configuration over a rather flat plateau containing, at a higher solvation, bicyclic [4.2.0] structures (**R6**, **R7**) corresponding to solid-state moieties isolated with tetramethylethylenediamine (TMEDA) (Figure S1B).^22^**B6** evolves toward more stable, less solvated species (**C4**, **C5**), with a higher number of Li–Cl bonds (Figures 1B, S2, and S3). Both **C4** and **C5** have cubane-like structures with Li’s and Cl’s at the vertices of two imbricated tetrahedra. The more symmetric **C4** arrangement has each Li bonded to three Cl’s, with distances of 2.43 ± 0.16 Å, and to one THF. It nicely resembles the solid-state structures of Li_4_Cl_4_(L)_4_, with L = Et_2_O and hexamethylphosphoramide (HMPA) (Table 1).^23,24^

The pentasolvated cluster, Li_4_Cl_4_(THF)_5_ (**C5**), is more stable than **C4** by 1.4 ± 0.6 kcal mol^–1^. Unlike **C4**, in **C5** one of the 12 Li–Cl bonds is broken, with a Li–Cl distance of 3.81 ± 0.03 Å. Two THFs solvate the dangling Li center, maintaining its tetrahedral coordination; the associated Cl passes from a μ_3_- to a μ_2_-bridging position between two proximate Li’s. Overall, **C5** shows larger flexibility than **C4**, with the root-mean-square fluctuations of the LiCl core increasing from 0.18 Å in **C4** to 0.34 Å in **C5**. The average bond distances and angles in **C5** are closer to those in Li_2_Cl_2_, indicating that cleaving a Li–Cl bond in **C4** relaxes the remaining part of the LiCl cluster (Figure S4). Thus, while no facile solvent-assisted LiCl bond cleavage occurs in Li_2_Cl_2_, the large solvated LiCl clusters appear to be more dynamic. Conversion of **C5** into **C4** requires a marginal activation energy (Δ*A*^⧧^ = 2.1 ± 0.8 kcal mol^–1^). On the contrary, there is essentially no energy barrier to open **C4** into **C5**, as confirmed by the systematic evolution of **C4** into **C5** observed within ∼5 ps of AIMD at room temperature. The identification of multiple structures for Li_4_Cl_4_ varying THF coordination evidences significant plasticity for this cluster, assisted by solvation.

We employed the global free energy minimum, **C5**, as the initial structure to investigate the interaction of LiCl with the monomeric forms, CH_3_MgCl, MgCl_2_, and Mg(CH_3_)_2_, of the Grignard reagent in the Schlenk equilibrium. Free energy estimates show that the binding affinity depends on the number of chlorides of the Grignard species (Figures 2 and S5–S7). Specifically, MgCl_2_ and Li_4_Cl_4_ bind as MgCl_2_·Li_4_Cl_4_ with a Δ*A* of −4.4 ± 1.1 kcal mol^–1^, while CH_3_MgCl shows only marginal affinity. In contrast, separated Mg(CH_3_)_2_ and Li_4_Cl_4_ are more stable than Mg(CH_3_)_2_·Li_4_Cl_4_ by ∼2 kcal mol^–1^.

**Figure 2:**
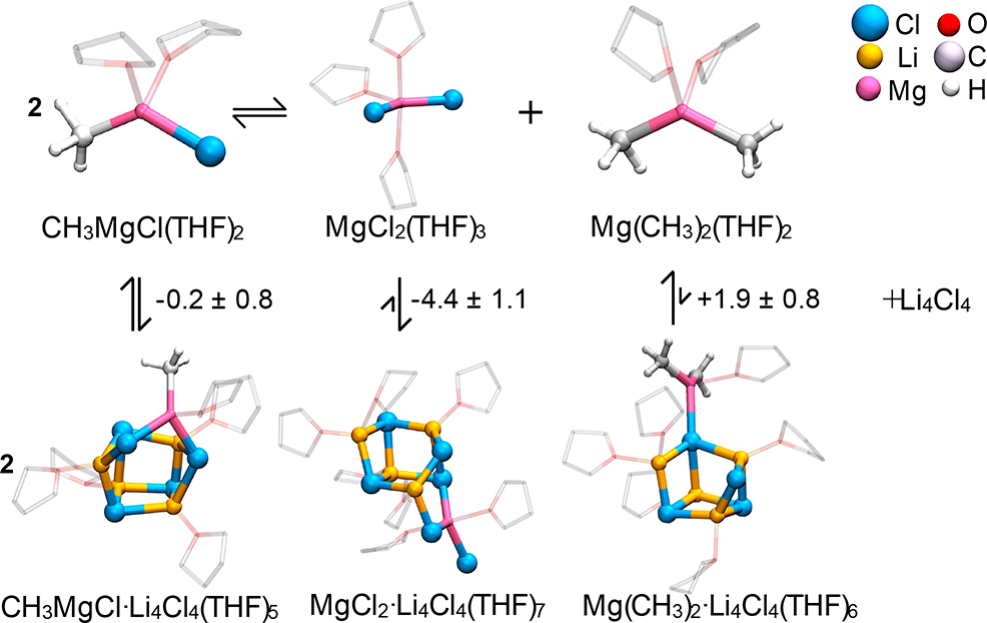
Interaction of the monomeric forms of the Grignard reagent with **C5**. Free energies are in kcal mol^–1^. THF hydrogens are not shown for clarity.

The interaction between Grignard and LiCl is triggered by the dangling lithium, which coordinates preferentially to the chloride of the Mg species, at the expense of expelling a THF from its coordination sphere. Subsequently, the μ_2_-Cl binds to the incoming Mg. Such generalized acid/base interactions are favored by the higher charges, negative at the μ_2_-Cl and positive at the open Li, relative to the other Li’s and Cl’s of the cluster (Figure S8). On the contrary, for Mg(CH_3_)_2_, Mg prefers to coordinate with a less electron-donating μ_3_-Cl, even if initially positioned nearby a μ_2_-Cl. This could be due to the stronger electron-donating effect of the methyl groups,^33^ enhancing repulsion with a more electron-rich chloride in the LiCl aggregate.

To evaluate the interactions between the Mg fragments and the LiCl cluster, the three Grignard–LiCl complexes were investigated by energy decomposition analysis (EDA)^37^ (Table S2). The interactions are mostly driven by the electrostatic term, with an additional charge-transfer contribution. These two components are the most stabilizing for MgCl_2_ and the least for Mg(CH_3_)_2_. This is in line with the high ionic character of the Mg–Cl bond that enhances both terms. The preference for Mg(CH_3_)_2_ to coordinate a weaker electron donor μ_3_-Cl agrees with the reduced Pauli repulsion between the two fragments.

The strongest binding of Li_4_Cl_4_ to MgCl_2_ has several implications. The energetic unbalance in favor of MgCl_2_·Li_4_Cl_4_ pushes the Schlenk equilibrium to the right, enhancing disproportionation of CH_3_MgCl and formation of the more electron-rich Mg(CH_3_)_2_.^34^ This does not exclude the occurrence of further equilibria, yielding other species. Another consequence is associated with the solvation pattern of Li_4_Cl_4_, influencing, in turn, its bonding properties. In CH_3_MgCl·Li_4_Cl_4_(THF)_5_ each metal is solvated by one THF, as in **C4**. On the contrary, in MgCl_2_·Li_4_Cl_4_(THF)_7_, both Mg and one of the Li’s are solvated by two THFs, similar to **C5**. The three ionic Mg–Cl bonds favor higher solvation of Mg as previously reported^25^ and account for its pentacoordination. Furthermore, the electron-withdrawing character of MgCl_2_ weakens the Li–Cl interactions, cleaving another Li–Cl bond with the assistance of additional solvation at Li (Figure 2 and Table S1).

To probe the tendency of MgCl_2_ to break down the LiCl cluster, we ran AIMD at a 1:1 LiCl:MgCl_2_ concentration, reporting a dramatic effect of MgCl_2_ on the compactness of LiCl. Upon binding, the newly formed bimetallic moiety undergoes rapid structural evolution, deforming the originally tight Li_4_Cl_4_ unit.

Relaxation of the cluster occurs through sequential cleavages of Li–Cl bonds and consequent opening of Li(μ-Cl)_2_Li rings (Figure 3). The LiCl moiety evolves along structures that closely recall those detected along the pathway of formation of Li_4_Cl_4_ (Figures 1B and S3), transitioning into an open cubane moiety resembling **R5**, a ladder conformation (**B6**), and a condensed [4.2.0] bicyclic entity (**R7**). In these structures, MgCl_2_ remains outside the decomposing Li_4_Cl_4_ cluster, connecting as Mg(μ_2_-Cl)_2_Li four-membered rings (Figure 3). Within 10 ps, the LiCl cluster reaches a structure comprising a single LiCl unit held on either side by homonuclear Li(μ_2_-Cl)_2_Li and mixed Mg(μ_2_-Cl)_2_Li rings (Figure 3). Such rapid evolution testifies to how the electron-withdrawing power of MgCl_2_ is sufficient to destroy the cohesive interactions within Li_4_Cl_4_. Moreover, the occurrence of a central, linear LiCl unit stabilized on both sides by small polynuclear Li:Mg:Cl aggregates suggests several scenarios. First, this exposed LiCl unit is more likely to participate in forthcoming reactions. Also, heterolytic cleavage of the central Li–Cl bond may be facilitated by the stabilizing effect of the terminal aggregates. Such an event could yield reactive ionic entities with active Li^+^ and Cl^–^ centers,^30^ despite a low dielectric solvent that would, per se, disfavor ionic separation.

**Figure 3:**
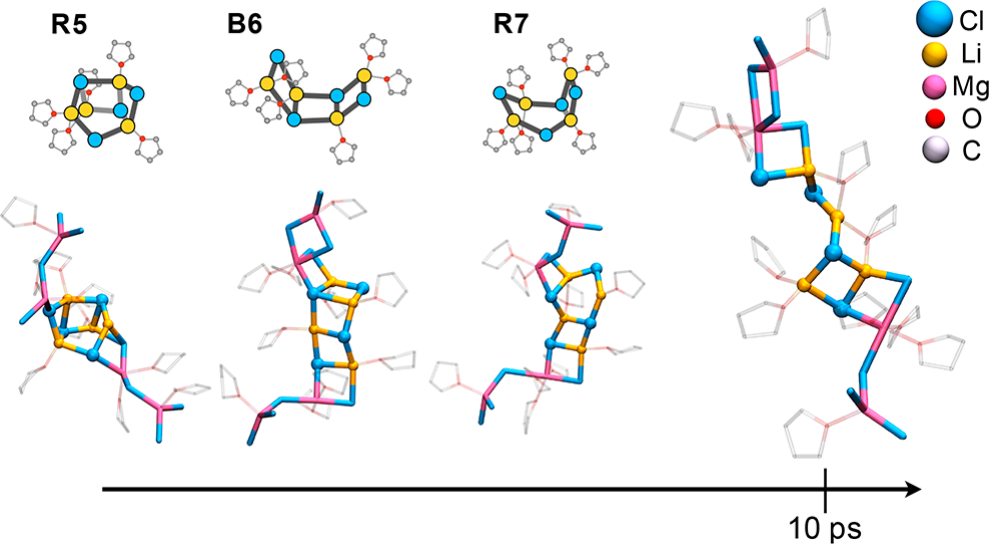
Time relaxation of solvated Li_4_Cl_4_ interacting with MgCl_2_ in a 1:1 Mg:Li ratio. Hydrogens are not shown for clarity.

AIMD employed a 1:1 LiCl:MgCl_2_ stoichiometry, while experiments typically use a 1:1 LiCl:RMgX ratio. Thus, the resulting LiCl:MgCl_2_ ratio should not exceed 2:1, assuming a quantitative shift of the Schlenk equilibrium induced by favorable LiCl–MgCl_2_ binding. Our computations were set up to favor conformational sampling in an affordable time for AIMD. Despite the excess concentration, the structures observed in simulations involved the direct interaction of Li_4_Cl_4_ with only two MgCl_2_ molecules, while other MgCl_2_ engaged peripherally through the dangling Mg–Cl bonds (Figure 3). Thus, the reported events are compatible with the experimentally more relevant 2:1 LiCl:MgCl_2_ ratio.

This study moves a critical step further into the comprehension of the chemistry of LiCl in ethereal solution and its interaction with Grignard species. LiCl emerges as a highly dynamic system whose selective affinity for MgCl_2_ deeply modifies the Schlenk equilibrium of Grignard reagents in favor of MgR_2_. Interaction with excess MgCl_2_ evolves the LiCl cluster toward a solubilization pathway that is otherwise energetically unfavorable. Solubilization involves transient structures related to those detected in crystalline samples. This confirms the existence of several energetically similar conformations for solvated LiCl, which may be stabilized by marginal changes in the environmental conditions.

Disintegration of Li_4_Cl_4_ by MgCl_2_ yields exposed LiCl moieties stabilized by small Li:Mg:Cl aggregates. Past experimental literature reported the presence of putative ionic species in turbo Grignard solutions.^30^ While a low-dielectric medium would disfavor the presence of charged compounds, the existence of nonbridging LiCl stabilized by the Li:Mg:Cl aggregates opens the possibility of forming Li^+^- and Cl^–^-based ionic compounds. These charged moieties could interact with the organomagnesium compounds present in solution, yielding other species of diverse reactivity. The LiCl-assisted enrichment of dialkylmagnesium confirms the original proposal by Knochel about its presence in solution,^2^ and it is also in line with its recent characterization as tBu_2_MgClLi(THF)_4_ in the solid state.^31^ This work urges further investigation on the fragmentation of Li:Mg:X clusters into neutral and ionic compounds as a function of R, halides, and solvent and on the consequences for turbo Grignard reagents.

## Data Availability

Simulation data presented in this work are openly available free of charge at the GitHub repository https://github.com/Cascella-Group-UiO/Publications.

## Supporting Information Available

Computational protocol, free energy profiles and associated constraint forces, molecular structures, molecular properties, and EDA analysis (PDF)

## References

[ref1] Krasovskiy A.; Knochel P. A LiCl-Mediated Br/Mg Exchange Reaction for the Preparation of Functionalized Aryl- and Heteroarylmagnesium Compounds from Organic Bromides. Angew. Chem., Int. Ed. 2004, 43, 3333–3336. 10.1002/anie.200454084. 15213967

[ref2] Krasovskiy A.; Straub B. F.; Knochel P. Highly Efficient Reagents for Br/Mg Exchange. Angew. Chem., Int. Ed. 2006, 45, 159–162. 10.1002/anie.200502220. 16307460

[ref3] Knochel P.; Gavryushin A.; Brade K. Functionalized Organomagnesium Compounds: Synthesis and Reactivity. In The Chemistry of Organomagnesium Compounds; Patai’s Chemistry of Functional Groups; Rappoport Z., Marek I., Eds.; John Wiley & Sons, 2009; pp 511–593.

[ref4] Li-Yuan Bao R.; Zhao R.; Shi L. Progress and Developments in the Turbo Grignard Reagent i-PrMgCl·LiCl: A Ten-Year Journey. Chem. Commun. 2015, 51, 6884–6900. 10.1039/C4CC10194D. 25714498

[ref5] Linton D. J.; Schooler P.; Wheatley A. E. H. Group 12 and Heavier Group 13 Alkali Metal ’Ate Complexes. Coord. Chem. Rev. 2001, 223, 53–115. 10.1016/S0010-8545(01)00379-4.

[ref6] Wheatley A. E. H. Recent Developments in the Synthetic and Structural Chemistry of Lithium Zincates. New J. Chem. 2004, 28, 435–443. 10.1039/b314042c.

[ref7] Krasovskiy A.; Krasovskaya V.; Knochel P. Mixed Mg/Li Amides of the Type R_2_NMgCl·LiCl as Highly Efficient Bases for the Regioselective Generation of Functionalized Aryl and Heteroaryl Magnesium Compounds. Angew. Chem., Int. Ed. 2006, 45, 2958–2961. 10.1002/anie.200504024. 16568481

[ref8] Campbell R.; García-Álvarez P.; Kennedy A. R.; Mulvey R. E. Synergic Transformation of an Ethylenediamine to a Lithium 1,3-Diaza-2-zincacyclopentene via an Alkyllithium/Bis(alkyl)zinc Mixture. Chem. - Eur. J. 2010, 16, 9964–9968. 10.1002/chem.201001314. 20645359 PMC3784041

[ref9] Campbell R.; Cannon D.; García-Álvarez P.; Kennedy A. R.; Mulvey R. E.; Robertson S. D.; Saßmannshausen J.; Tuttle T. Main Group Multiple C–H/N–H Bond Activation of a Diamine and Isolation of A Molecular Dilithium Zincate Hydride: Experimental and DFT Evidence for Alkali Metal–Zinc Synergistic Effects. J. Am. Chem. Soc. 2011, 133, 13706–13717. 10.1021/ja205547h. 21777000 PMC3662402

[ref10] Wang Y.; Xie Y.; Abraham M. Y.; Gilliard R. J.; Wei P.; Campana C. F.; Schaefer H. F.; Schleyer P. v. R.; Robinson G. H. NHC-Stabilized Triorganozincates: Syntheses, Structures, and Transformation to Abnormal Carbene-Zinc Complexes. Angew. Chem., Int. Ed. 2012, 51, 10173–10176. 10.1002/anie.201204712. 22962055

[ref11] Armstrong D. R.; Crosbie E.; Hevia E.; Mulvey R. E.; Ramsay D. L.; Robertson S. D. TMP (2,2,6,6-Tetramethylpiperidide)-Aluminate Bases: Lithium-Mediated Alumination or Lithiation–Alkylaluminium-Trapping Reagents?. Chem. Sci. 2014, 5, 3031–3045. 10.1039/C4SC01108B.

[ref12] Tejo C.; Pang J. H.; Ong D. Y.; Oi M.; Uchiyama M.; Takita R.; Chiba S. Dearylation of Arylphosphine Oxides Using a Sodium Hydride–Iodide Composite. Chem. Commun. 2018, 54, 1782–1785. 10.1039/C8CC00289D. 29383363

[ref13] Robertson S. D.; Uzelac M.; Mulvey R. E. Alkali-Metal-Mediated Synergistic Effects in Polar Main Group Organometallic Chemistry. Chem. Rev. 2019, 119, 8332–8405. 10.1021/acs.chemrev.9b00047. 30888154

[ref14] Schlenk W.; Schlenk W. Über die Konstitution der Grignardschen Magnesiumverbindungen. Ber. Dtsch. Chem. Ges. 1929, 62, 920–924. 10.1002/cber.19290620422.

[ref15] Walker F. W.; Ashby E. C. Composition of Grignard Compounds. VI. Nature of Association in Tetrahydrofuran and Diethyl Ether Solutions. J. Am. Chem. Soc. 1969, 91, 3845–3850. 10.1021/ja01042a027.

[ref16] Ashby E. C.; Nackashi J.; Parris G. E. Composition of Grignard Compounds. X. NMR, IR and Molecular Association Studies of Some Methylmagnesium Alkoxides in Diethyl Ether, Tetrahydrofuran, and Benzene. J. Am. Chem. Soc. 1975, 97, 3162–3171. 10.1021/ja00844a040.

[ref17] Seyferth D. The Grignard Reagents. Organometallics 2009, 28, 1598–1605. 10.1021/om900088z.

[ref18] Raston C. L.; Skelton B. W.; Whitaker C. R.; White A. H. Lewis-Base Adducts of Main Group-1 Metal-Compounds. IX. Synthesis and Structural Characterization of Adducts of the Lithium(1) Halides With N,N,N’,N’-Tetramethylethylenediamine. Aust. J. Chem. 1988, 41, 1925–1934. 10.1071/CH9881925.

[ref19] Bauer S. H.; Ino T.; Porter R. F. Molecular Structure of Lithium Chloride Dimer. Thermodynamic Functions of Li_2_X_2_ (X = Cl, Br, I). J. Chem. Phys. 1960, 33, 685–691. 10.1063/1.1731238.

[ref20] Schmuck A.; Leopold D.; Wallenhauer S.; Seppelt K. Strukturen von Pentaarylbismut-Verbindungen. Chem. Ber. 1990, 123, 761–766. 10.1002/cber.19901230419.

[ref21] Hahn F. E.; Rupprecht S. Synthese und Kristallstruktur von [LiCl·2THF]_2_/Synthesis and Crystal Structure of [LiCl·2THF]_2_. Z. Naturforsch. B 1991, 46, 143–146. 10.1515/znb-1991-0203.

[ref22] Hoffmann D.; Dorigo A.; von Schleyer P.; Reif H.; Stalke D.; Sheldrick G. M.; Weiss E.; Geissler M. The Bicyclic Structure of a Novel TMEDA-Solvated Lithium Chloride Tetramer [(LiCl)_4_·3.5TMEDA]_2_: X-ray Structural Analysis and MO Investigations. Inorg. Chem. 1995, 34, 262–269. 10.1021/ic00105a042.

[ref23] Barr D.; Clegg W.; Mulvey R. E.; Snaith R. Crystal Structures of (Ph_2_C=NLi·NC_5_H_5_)_4_ and [CILi·O=P(NMe_2_)_3_]_4_; Discrete Tetrameric Pseudo-Cubane Clusters with Bridging of Li_3_ Triangles by Nitrogen and by Chlorine A. J. Chem. Soc., Chem. Commun. 1984, 79–80. 10.1039/C39840000079.

[ref24] Mitzel N. W.; Lustig C. Notizen: Crystal Structure of a Lithium Chloride Cubane Cluster Solvated by Diethyl Ether. Z. Naturforsch. B 2001, 56, 443–445. 10.1515/znb-2001-4-521.

[ref25] Durant F.; Gobillon Y.; Piret P.; van Meerssche M. Étude Par Diffraction de Rayons X de Complexes D’Halogénures Alcalins et de Molécules Organiques. V. Structure Du Complexe Chlorure Du Lithium·Dioxanne-1,4. Bull. Soc. Chim. Bel. 1966, 75, 52–69. 10.1002/bscb.19660750106.

[ref26] Kopp M. R.; Neumüller B. Die Kristallstruktur von [(THF)LiCl]_*n*_/The Crystal Structure of [(THF)LiCl]_*n*_. Z. Naturforsch. B 1999, 54, 818–820. 10.1515/znb-1999-0619.

[ref27] Hope H.; Oram D.; Power P. P. Isolation and X-Ray Crystal Structure of the Cuprate Complex Decaethoxydichlorotetralithium Bis(Hexaphenyldilithiumtricuprate) ([Li_2_Cu_3_Ph_6_]_2_[Li_4_Cl_2_(Et_2_O)_10_]): The First X-ray Structural Characterization of an Anionic Organocopper-Lithium Cluster. J. Am. Chem. Soc. 1984, 106, 1149–1150. 10.1021/ja00316a073.

[ref28] Buttrus N. H.; Eaborn C.; Hitchcock P. B.; Smith J. D.; Stamper J. G.; Sullivan A. C. The Crystal Structure of [(PMDETA)Li(μ-Cl)Li(PMDETA)][Li{C(SiMe_3_)_3_}_2_] [PMDETA = Me_2_N(CH_2_)_2_NMe(CH_2_)_2_NMe_2_]. A Novel Linear Chlorine-Centred Cation. J. Chem. Soc., Chem. Commun. 1986, 969–970. 10.1039/C39860000969.

[ref29] Blasberg F.; Bolte M.; Wagner M.; Lerner H.-W. An Approach to Pin Down the Solid-State Structure of the “Turbo Grignard. Organometallics 2012, 31, 1001–1005. 10.1021/om201080t.

[ref30] Schnegelsberg C.; Bachmann S.; Kolter M.; Auth T.; John M.; Stalke D.; Koszinowski K. Association and Dissociation of Grignard Reagents RMgCl and Their Turbo Variant RMgCl·LiCl. Chem. - Eur. J. 2016, 22, 7752–7762. 10.1002/chem.201600699. 27150118

[ref31] Hermann A.; Seymen R.; Brieger L.; Kleinheider J.; Grabe B.; Hiller W.; Strohmann C. Comprehensive Study of the Enhanced Reactivity of Turbo-Grignard Reagents. Angew. Chem., Int. Ed. 2023, 62, e202302489 10.1002/anie.202302489. 36971042

[ref32] Car R.; Parrinello M. Unified Approach for Molecular Dynamics and Density-Functional Theory. Phys. Rev. Lett. 1985, 55, 2471–2474. 10.1103/PhysRevLett.55.2471. 10032153

[ref33] Peltzer R. M.; Eisenstein O.; Nova A.; Cascella M. How Solvent Dynamics Controls the Schlenk Equilibrium of Grignard Reagents: A Computational Study of CH_3_MgCl in Tetrahydrofuran. J. Phys. Chem. B 2017, 121, 4226–4237. 10.1021/acs.jpcb.7b02716. 28358509

[ref34] Peltzer R. M.; Gauss J.; Eisenstein O.; Cascella M. The Grignard Reaction – Unraveling a Chemical Puzzle. J. Am. Chem. Soc. 2020, 142, 2984–2994. 10.1021/jacs.9b11829. 31951398

[ref35] Edwards A. J.; Paver M. A.; Raithby P. R.; Russell C. A.; Wright D. S. Dalton Communications. The ‘Broken Cube’ Polymer Structure of (LiBr·thf)_∞_ (Thf = Tetrahydrofuran). J. Chem. Soc., Dalton Trans. 1993, 3265–3266. 10.1039/DT9930003265.

[ref36] Chivers T.; Downard A.; Parvez M.; Schatte G. The Many Guises of Lithium Chloride: Crystal Structure of the Single-Strand Polymer {LiCl·2MeCN}_∞_. Inorg. Chem. 2001, 40, 1975–1977. 10.1021/ic001175c. 11312757

[ref37] Bickelhaupt F. M.; Baerends E. J. Kohn-Sham Density Functional Theory: Predicting and Understanding Chemistry. Rev. Comput. Chem. 2000, 15, 1–86. 10.1002/9780470125922.ch1.

