# Supplementary material for: Morphological Plasticity of LiCl Clusters Interacting with Grignard Reagent in Tetrahydrofuran
Source: J Am Chem Soc. 2023 Jul 20;145(30):16305–9. doi: 10.1021/jacs.3c04238 (PMC10401704; doi:10.1021/jacs.3c04238)
Supplement: Supplementary file 1 — ja3c04238_si_001.pdf [file ja3c04238_si_001.pdf]

Supporting Information for:

Morphological plasticity of LiCl clusters interacting with Grignard reagent in tetrahydrofuran

Marinella de Giovanetti,[†] Sondre H. Hopen Eliasson,[†] Abril C. Castro,[†] Odile Eisenstein,[*‡,†] Michele Cascella[*†]

[†]Department of Chemistry and Hylleraas Centre for Quantum Molecular Sciences, University of Oslo, PO Box 1033 Blindern, 0315 Oslo, Norway
[‡]ICGM, University of Montpellier; CNRS, ENSCM, 34090 Montpellier, France

Table of contents

1. Computational methods S2
2. Li_4X_4 (X = Cl and Br) structures observed experimentally and calculated (X = Cl) in this work S4
3. Free energy profiles for Li_4Cl_4 as a function of the solvation S5
4. Calculated structural parameters of LiCl species S6
5. Free energy profiles for Grignard species interacting with Li_4Cl_4 S6
6. Atomic charges of LiCl and associated Grignard·LiCl species S8
7. Calculated structural parameters of selected clusters. S9
8. Energy decomposition analysis of selected clusters. S10

1. Computational methods

AIMD Simulations

The initial model system, created using the PACKMOL package,[1] consists of a cubic periodic box of 20 Å length filled by THF molecules to reproduce the experimental density of THF at room temperature.[2] The followed protocol is analogous to what described in previous works.[3,4] The electronic problem is solved at the DFT level of theory, with the PBE approximation of the exchange-correlation functional,[5] and D3 Grimme correction for dispersion.[6] The choice of the PBE functional is based on benchmark calculations presented in ref.[4] All atoms are described with a DZVP basis set, using a molecularly optimized version for Li and Cl, and with GTH pseudopotentials. Auxiliary plane-waves basis set is cut-off to 250 Ry. AIMD simulations are run in the NVT ensemble using the Velocity Verlet algorithm with 0.25 fs timestep, fixing the temperature at 300K with a CSVR thermostat[7] of 50 ps time constant. AIMD trajectories are computed with the CP2K package[8] and analyzed with the VMD 1.9.4 software.[9] The initial coordinates are obtained following a 30 ps run at the target temperature to ensure equilibration of the system.

Free energy calculations

Free energy surfaces are computed by thermodynamic integration in the Blue Moon ensemble:[10]

$$\Delta A = \int \langle F_c(\lambda) \rangle \, d\lambda \qquad (S1)$$

where λ is a generic collective variable.

Li_4Cl_4: λ = coordination number (CN)[11] between Li and O(THF), estimated by:

$$CN[X](t) = \sum_{i}^{N_{Li}} \sum_{j}^{N_O} \frac{1 - \left(\frac{d_{ij}(t)}{R_0} \right)^n}{1 - \left(\frac{d_{ij}(t)}{R_0} \right)^m} \qquad (S2)$$

where n = 12, m = 24, R_0 = 2.5Å and d_{ij} is the distance between each Li and O.

Grignard + Li_4Cl_4: λ = distance (d) between the two species:

- CH_3MgCl – Distance between the midpoints of the Mg-Cl and the open-Li-Cl bonds,
- $MgCl_2$ – Distance between Mg and the center of mass of Li_4Cl_4,
- $Mg(CH_3)_2$ – Distance between Mg and μ_2-Cl.

The constraint force F_c is collected over 10-30 ps runs, monitoring the running average as convergence criterium[12] (discarding the first 5 ps of the trajectory to ensure equilibration at the target value). All graphs report the standard deviation of the constraint forces collected over AIMD trajectories as error bars. The uncertainties on the free energy points are estimated by propagation of the error from the standard deviation of the forces.

Energy Decomposition Analysis

$CH_3MgCl{\cdot}Li_4Cl_4(THF)_5$, $MgCl_2{\cdot}Li_4Cl_4(THF)_7$, and $Mg(CH_3)_2{\cdot}Li_4Cl_4(THF)_6$ were optimized at the PBE-D3/TZ2P level, [5] using the COSMO model[13,14] for simulating bulk solvation in THF. These structures were used to perform a quantitative energy decomposition analysis (EDA)[15] as implemented in the ADF software package.[16,17] We selected the monomeric forms of the Grignard

reagent (CH_3MgCl, $MgCl_2$, and $Mg(CH_3)_2$) and the Li_4Cl_4 as fragments in order to explore the Mg–(LiCl) interactions.

Quantitative EDA used in this work divides the interaction energy ΔE_{int} into four physically meaningful terms:

$$\Delta E_{int} = \Delta V_{elstat} + \Delta E_{Pauli} + \Delta E_{orb} + \Delta E_{disp} \tag{S3}$$

The classical electrostatic interaction ΔV_{elstat} is the energy between the unperturbed charge distributions of the prepared fragments. The Pauli repulsion ΔE_{Pauli}, responsible for steric repulsion, arises from the destabilizing interactions between occupied orbitals of the fragments. The stabilizing orbital interaction term ΔE_{orb} accounts for charge transfer and polarization, and the dispersion energy ΔE_{disp} is a long-range electron correlation effect.

2. Li_4X_4 (X = Cl and Br) structures observed experimentally and calculated (X = Cl) in this work

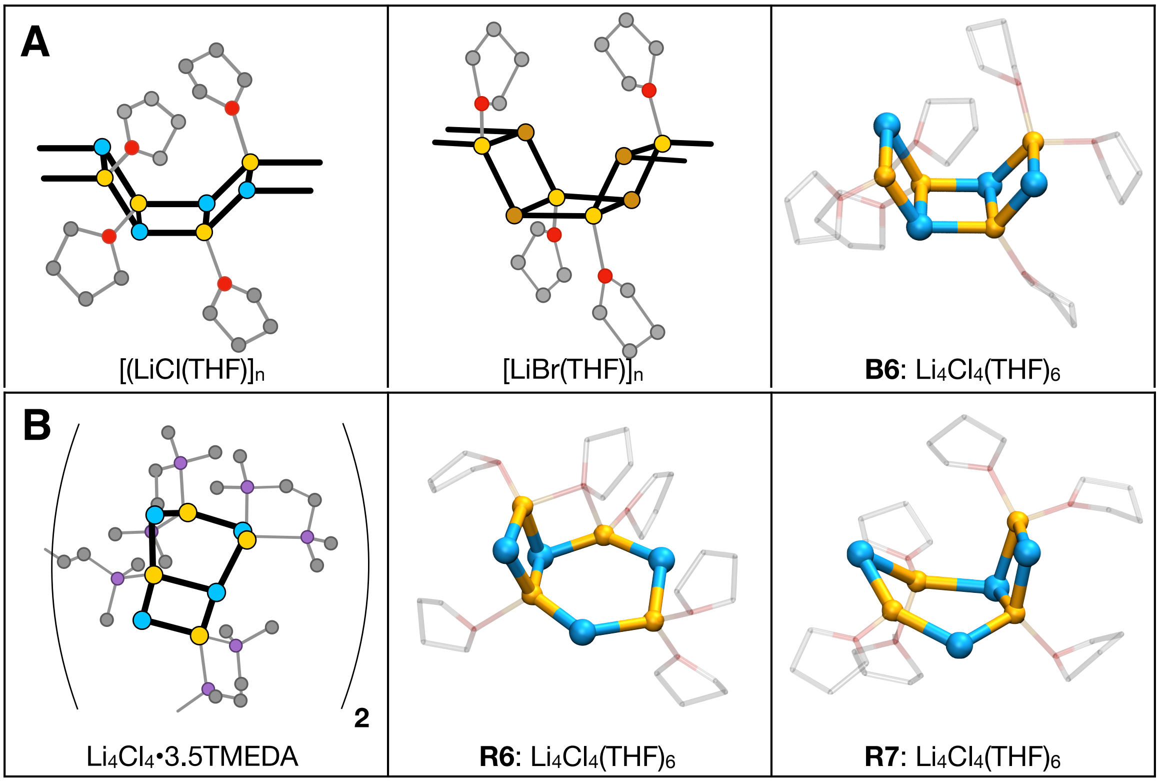

Figure S1. Panel A: polymeric LiCl[18] and LiBr[19] identified experimentally and $Li_4Cl_4(THF)_6$ aggregate (**B6**) obtained in this work. Panel B: bicyclic [4:2:0] LiCl[20] identified experimentally and bicyclic $Li_4Cl_4(THF)_6$ aggregates (**R6**, **R7**) obtained in this work. Blue = Cl, brown = Br, yellow = Li, red = O, purple = N, grey = C. Hydrogens are not shown for clarity.

3. Free energy profiles for Li_4Cl_4 as a function of the solvation

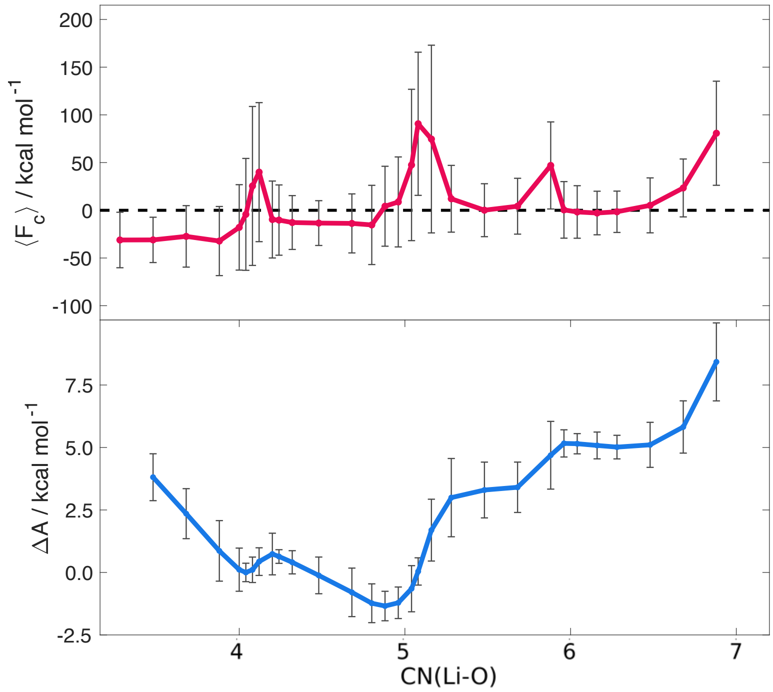

Figure S2. Constraint force (top) and reconstructed free energy surface (bottom) for tetrameric Li_4Cl_4, as a function of the average coordination number between Li and O(THF).

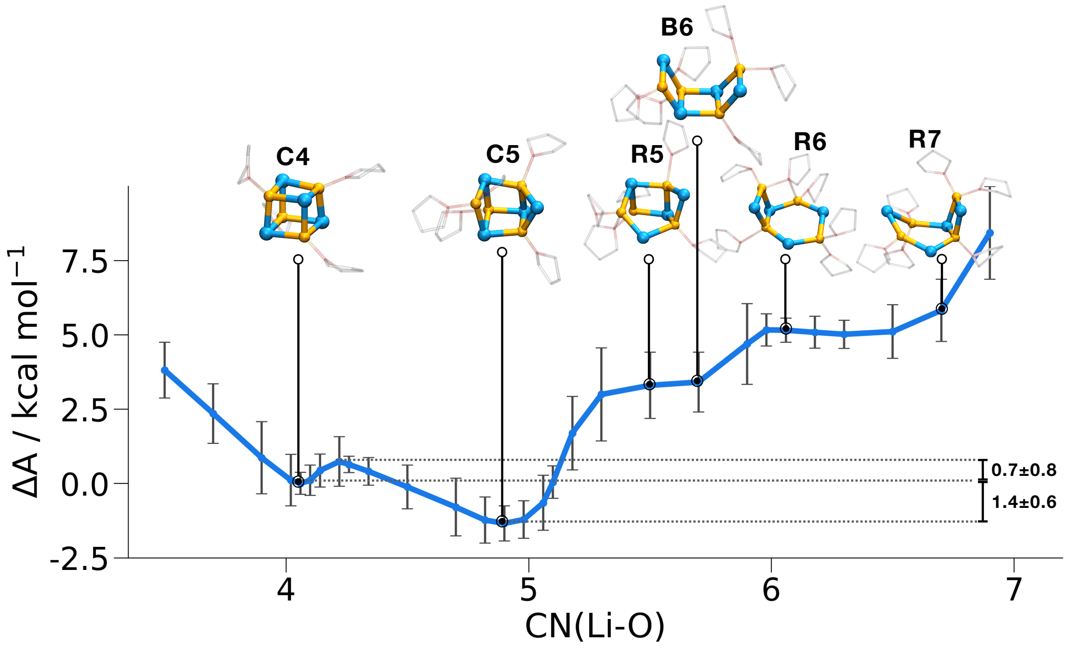

Figure S3. Li_4Cl_4 structures obtained in this work represented on the reconstructed free energy surface for tetrameric Li_4Cl_4. Blue = Cl, yellow = Li, red = O, grey = C. Hydrogens are not shown for clarity.

4. Calculated structural parameters of LiCl species

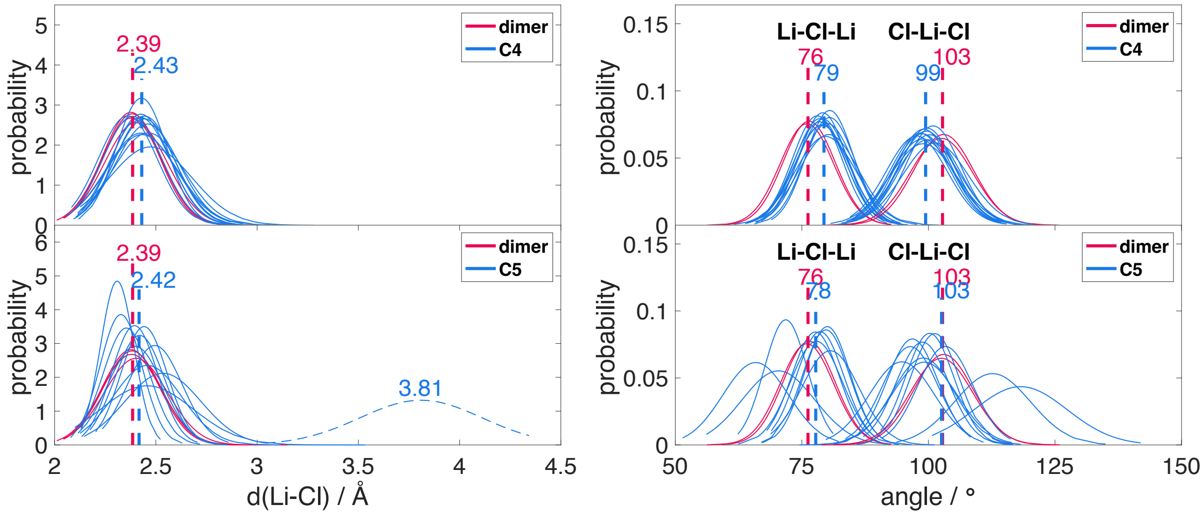

Figure S4. Bond and angle distributions of dimeric $Li_2Cl_2(THF)_4$, closed cubane (**C4**), and semi-open cubane (**C5**) Li_4Cl_4 structures. See figure S3 for labels.

5. Free energy profiles for Grignard species interacting with Li_4Cl_4

a. CH_3MgCl

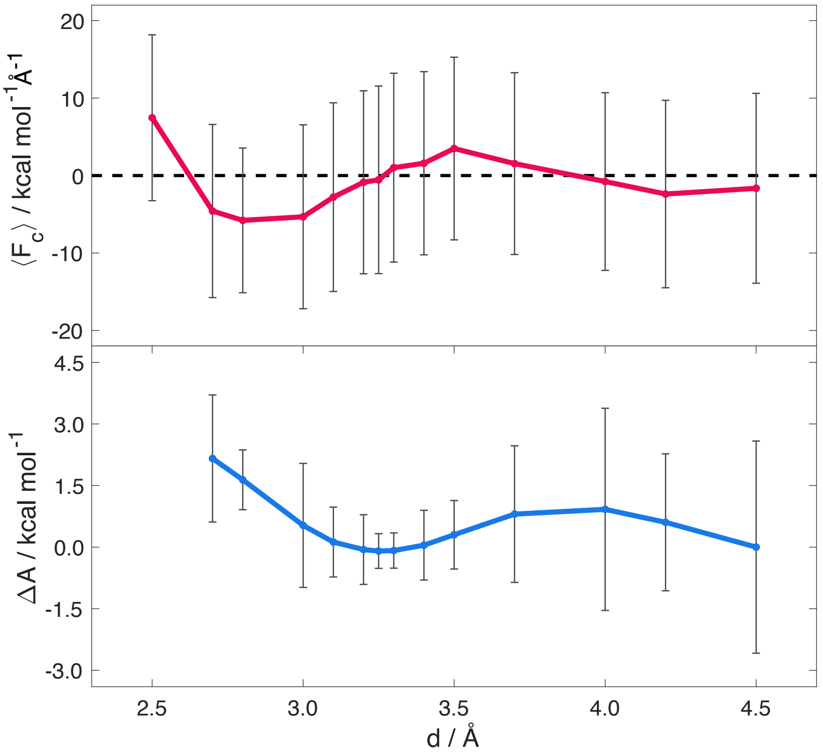

Figure S5. Constraint force (top) and reconstructed free energy surface (bottom) for the formation of $CH_3MgCl{\cdot}Li_4Cl_4$.

b. $MgCl_2$

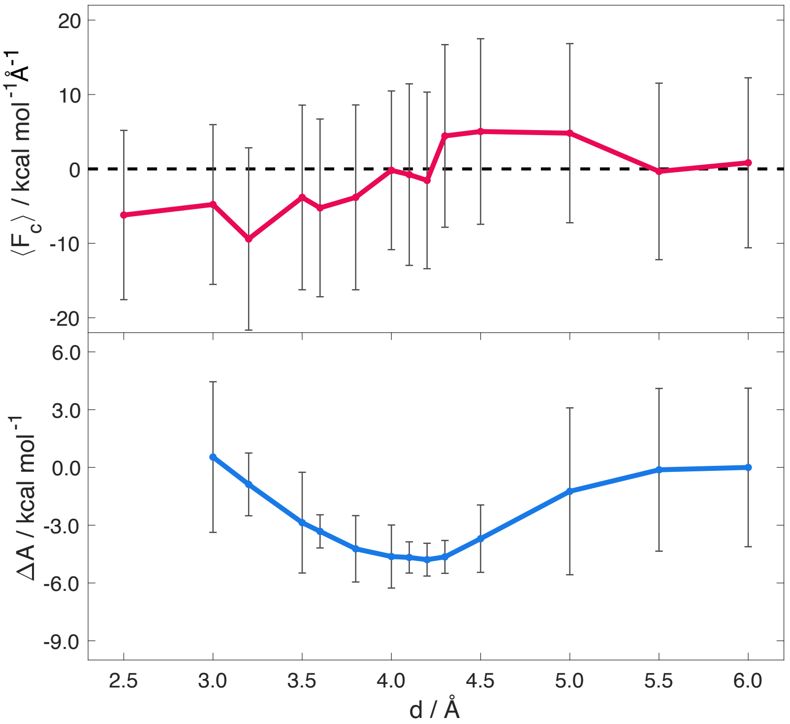

Figure S6. Constraint force (top) and reconstructed free energy surface (bottom) for the formation of $MgCl_2 \cdot Li_4Cl_4$.

c. **$Mg(CH_3)_2$**

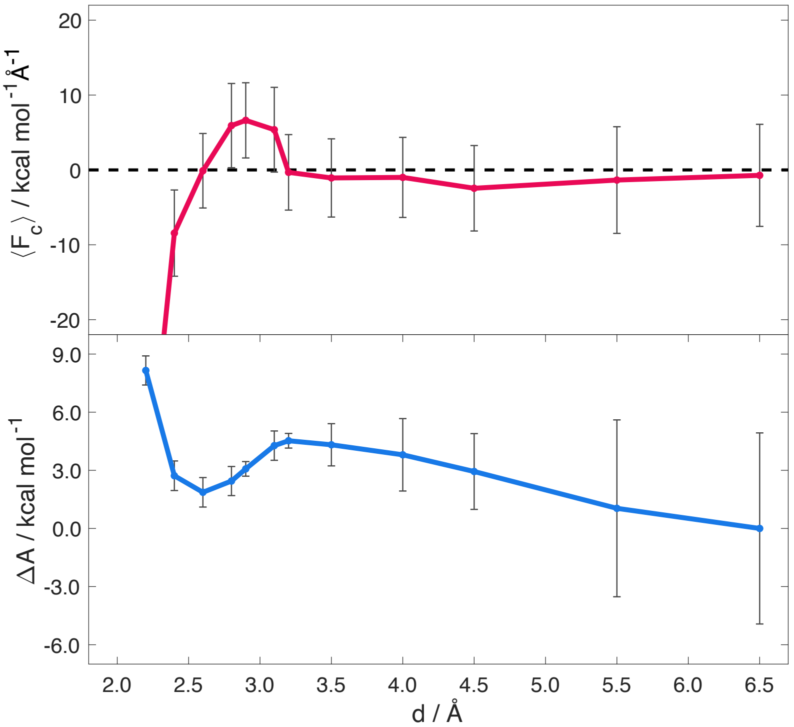

Figure S7. Constraint force (top) and reconstructed free energy surface (bottom) for the formation of $Mg(CH_3)_2 \cdot Li_4Cl_4$.

6. Atomic charges of LiCl and associated Grignard·LiCl species

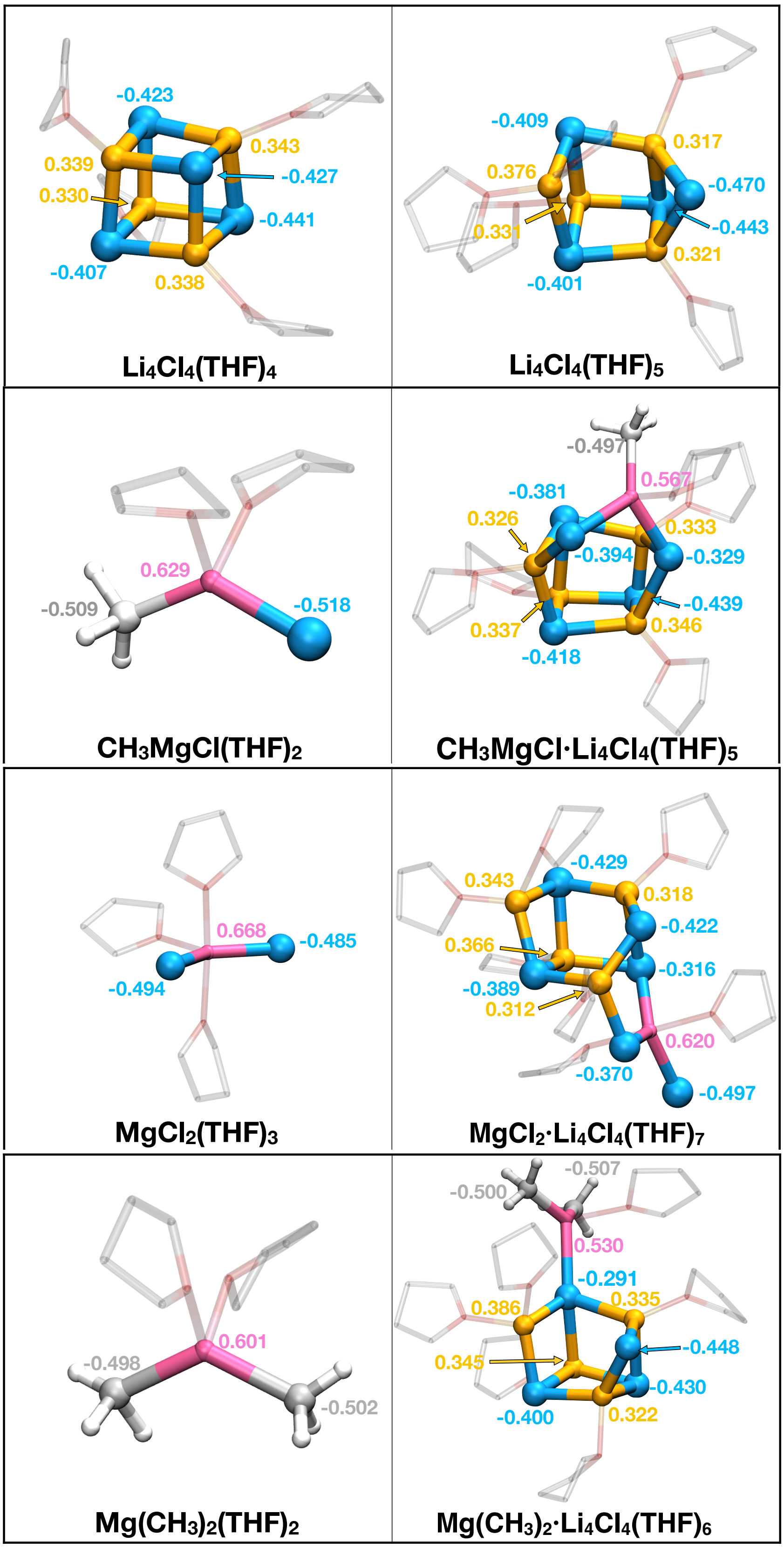

Figure S8. CM5 atomic charges[21] for the compounds investigated in this work. The charges are obtained as averages for snapshots taken every 125 fs over the NVT trajectory. Blue = Cl, yellow = Li, mauve = Mg, red = O, grey = C. THF hydrogens are not shown for clarity.

7. Calculated structural parameters of selected clusters

Table S1. Structural parameters of Grignard and Grignard-LiCl species, obtained from NVT runs. Bond distances are given in Å and angles in deg. X,Y = CH_3 or Cl. When bonds or angles involve chemically non-equivalent Cl, data is listed in order of increasing coordination at the Cl (µ1 →µ2→µ3).

	d(Mg-C)	d(Mg-Cl)	X-Mg-Y	X-Mg-X
$CH_3MgCl(THF)_2$	2.14(5)	2.35(6)	128(11)	-
$CH_3MgCl\cdot Li_4Cl_4(THF)_5$	2.14(6)	2.43(8)<br>2.48(9)	119(9)<br>127(8)	103(7)
$MgCl_2(THF_3)$	-	2.32(11)	-	128(9)
$MgCl_2\cdot Li_4Cl_4(THF)_7$	-	2.36(7)<br>2.39(8)<br>2.44(9)	-	118(7)<br>126(10)<br>112(7)
$Mg(CH_3)_2(THF)_2$	2.17(7)	-	-	135(10)
$Mg(CH_3)_2\cdot Li_4Cl_4(THF)_6$	2.17(7)	2.67(16)	108(9)	131(9)

8. Energy decomposition analysis of selected clusters

Table S2. Energy Decomposition Analysis (EDA) of $CH_3MgCl·Li_4Cl_4(THF)_5$, $MgCl_2·Li_4Cl_4(THF)_7$, and $Mg(CH_3)_2·Li_4Cl_4(THF)_6$, computed at the PBE-D3/TZ2P level. All energies are in kcal mol^{-1}. The fragments were selected considering the interaction between the Grignard moiety (CH_3MgCl, $MgCl_2$, or $Mg(CH_3)_2$) and the Li_4Cl_4 fragment.[a]

[a] The fragments are kept in the frozen geometry of the compound and thus there is no preparation energy term.

[b] Total interaction energy defined as in equation S3.

[c] Selecting Cl(1) atom for the CH_3MgCl fragment.

[d] Selecting Cl(2) atom for the CH_3MgCl fragment.

[e] Selecting Cl(1) atom for the $MgCl_2$ fragment.

[f] Selecting Cl(2) atom for the $MgCl_2$ fragment.

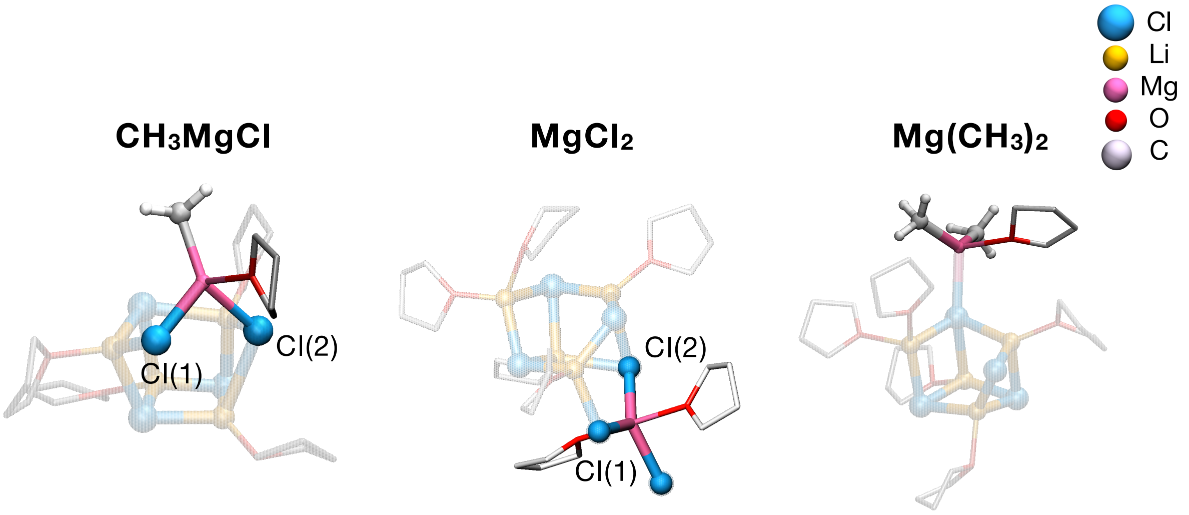

	CH_3MgCl		$MgCl_2$		$Mg(CH_3)_2$
	Cl(1)[c]	Cl(2)[d]	Cl(1)[e]	Cl(2)[f]	
Total interaction (ΔE_{int})[b]	-55.3	-79.5	-60.5	-89.0	-31.9
Pauli (ΔE_{Pauli})	47.7	64.1	53.8	67.4	34.8
Electrostatic (ΔV_{elstat})	-62.8 (68.2 %)	-89.3 (67.7 %)	-67.2 (68.4 %)	-95.6 (68.4 %)	-36.2 (67.7%)
Orbital (ΔE_{orb})	-29.3 (31.8 %)	-42.6 (32.3 %)	-31.1 (31.6 %)	-44.3 (31.6 %)	-17.3 (32.3 %)
Dispersion (ΔE_{disp})	-10.9	-11.8	-16.0	-16.5	-13.2

References

(1) Martínez, L.; Andrade, R.; Birgin, E. G.; Martínez, J. M. PACKMOL: A Package for Building Initial Configurations for Molecular Dynamics Simulations. *J. Comput. Chem.* **2009**, *30*, 2157–2164.

(2) Metz, D. J.; Glines, A. Density, Viscosity, and Dielectric Constant of Tetrahydrofuran between -78 and 30.Degree. *J. Phys. Chem.* **1967**, *71*, 1158–1158..

(3) Peltzer, R. M.; Eisenstein, O.; Nova, A.; Cascella, M. How Solvent Dynamics Controls the Schlenk Equilibrium of Grignard Reagents: A Computational Study of CH_3MgCl in Tetrahydrofuran. *J. Phys. Chem. B* **2017**, *121*, 4226–4237.

(4) Peltzer, R. M.; Gauss, J.; Eisenstein, O.; Cascella, M. The Grignard Reaction – Unraveling a Chemical Puzzle. *J. Am. Chem. Soc.* **2020**, *142*, 2984–2994.

(5) Perdew, J. P.; Burke, K.; Ernzerhof, M. Generalized Gradient Approximation Made Simple. *Phys. Rev. Lett.* **1996**, *77*, 3865–3868..

(6) Grimme, S.; Antony, J.; Ehrlich, S.; Krieg, H. A Consistent and Accurate Ab Initio Parametrization of Density Functional Dispersion Correction (DFT-D) for the 94 Elements H-Pu. *J. Chem. Phys.* **2010**, *132*, 154104.

(7) Bussi, G.; Donadio, D.; Parrinello, M. Canonical Sampling through Velocity Rescaling. *J. Chem. Phys.* **2007**, *126*, 014101.

(8) Kühne, T. D.; Iannuzzi, M.; Del Ben, M.; Rybkin, V. V.; Seewald, P.; Stein, F.; Laino, T.; Khaliullin, R. Z.; Schütt, O.; Schiffmann, F.; Golze, D.; Wilhelm, J.; Chulkov, S.; Bani-Hashemian, M. H.; Weber, V.; Borštnik, U.; Taillefumier, M.; Jakobovits, A. S.; Lazzaro, A.; Pabst, H.; Müller, T.; Schade, R.; Guidon, M.; Andermatt, S.; Holmberg, N.; Schenter, G. K.; Hehn, A.; Bussy, A.; Belleflamme, F.; Tabacchi, G.; Glöß, A.; Lass, M.; Bethune, I.; Mundy, C. J.; Plessl, C.; Watkins, M.; VandeVondele, J.; Krack, M.; Hutter, J. CP2K: An Electronic Structure and Molecular Dynamics Software Package - Quickstep: Efficient and Accurate Electronic Structure Calculations. *J. Chem. Phys.* **2020**, *152*, 194103.

(9) Humphrey, W.; Dalke, A.; Schulten, K. VMD: Visual Molecular Dynamics. *J. Mol. Graph.* **1996**, *14*, 33–38.

(10) Carter, E. A.; Ciccotti, G.; Hynes, J. T.; Kapral, R. Constrained Reaction Coordinate Dynamics for the Simulation of Rare Events. *Chem. Phys. Lett.* **1989**, *156*, 472–477.

(11) Iannuzzi, M.; Laio, A.; Parrinello, M. Efficient Exploration of Reactive Potential Energy Surfaces Using Car-Parrinello Molecular Dynamics. *Phys. Rev. Lett.* **2003**, *90*, 238302.

(12) Isayev, O.; Gorb, L.; Leszczynski, J. Theoretical Calculations: Can Gibbs Free Energy for Intermolecular Complexes Be Predicted Efficiently and Accurately? *J. Comput. Chem.* **2007**, *28*, 1598–1609.

(13) Klamt, A.; Schüürmann, G. COSMO: A New Approach to Dielectric Screening in Solvents with Explicit Expressions for the Screening Energy and Its Gradient. *J. Chem. Soc., Perkin Trans. 2* **1993**, No. 5, 799–805.

(14) Pye, C. C.; Ziegler, T. An Implementation of the Conductor-like Screening Model of Solvation within the Amsterdam Density Functional Package. *Theor. Chem. Accounts Theory, Comput. Model. (Theoretica Chim. Acta)* **1999**, *101*, 396–408.

(15) Bickelhaupt, F. M.; Baerends, E. J. Kohn-Sham Density Functional Theory: Predicting and Understanding Chemistry; In: *Reviews in Computational Chemistry, vol. **15***. Lipkowitz, K. B.; Boyd, B. D. eds. Wiley-VCH 2000; pp 1–86.

(16) te Velde, G.; Bickelhaupt, F. M.; Baerends, E. J.; Fonseca Guerra, C.; van Gisbergen, S. J. A.; Snijders, J. G.; Ziegler, T. Chemistry with ADF. *J. Comput. Chem.* **2001**, *22*, 931–967.

(17) Baerends, E.-J. et al., ADF Program. In *SCM, Theoretical Chemistry*; Vrije Universiteit: Amsterdam, The Netherlands, 2023.

(18) Kopp, M. R.; Neumüller, B. Die Kristallstruktur von $[(THF)LiCl]_n$ / The Crystal Structure of $[(THF)LiCl]_N$. *Z. Naturforsch. B* **1999**, *54*, 818–820.
(19) Edwards, A. J.; Paver, M. A.; Raithby, P. R.; Russell, C. A.; Wright, D. S. Dalton Communications. The 'Broken Cube' Polymer Structure of $(LiBr{\cdot}thf)_\infty$ (Thf = Tetrahydrofuran). *J. Chem. Soc., Dalt. Trans.* **1993**, 3265–3266.
(20) Hoffmann, D.; Dorigo, A.; von Schleyer, P.; Reif, H.; Stalke, D.; Sheldrick, G. M.; Weiss, E.; Geissler, M. The Bicyclic Structure of a Novel TMEDA-Solvated Lithium Chloride Tetramer $[(LiCl)_4{\cdot}3.5TMEDA]_2$: X-Ray Structural Analysis and MO Investigations. *Inorg. Chem.* **1995**, *34*, 262–269.
(21) Marenich, A. V.; Jerome, S. V.; Cramer, C. J.; Truhlar, D. G. Charge Model 5: An Extension of Hirshfeld Population Analysis for the Accurate Description of Molecular Interactions in Gaseous and Condensed Phases. *J. Chem. Theory Comput.* **2012**, *8*, 527–541.
